# Prediction of Cognitive Decline from White Matter Hyperintensity and Single-Photon Emission Computed Tomography in Alzheimer’s Disease

**DOI:** 10.3389/fneur.2017.00408

**Published:** 2017-09-04

**Authors:** Ken-ichi Tabei, Hirotaka Kida, Tetsuo Hosoya, Masayuki Satoh, Hidekazu Tomimoto

**Affiliations:** ^1^Department of Dementia Prevention and Therapeutics, Graduate School of Medicine, Mie University, Mie, Japan; ^2^Department of Neurology, Graduate School of Medicine, Mie University, Mie, Japan; ^3^FUJIFILM RI Pharma Co., Ltd., Tokyo, Japan

**Keywords:** cognitive decline, dementia, Alzheimer’s disease, white matter hyperintensity, single-photon emission computed tomography, neuropsychological test

## Abstract

**Background:**

While several studies support an association of white matter hyperintensity (WMH) volume and regional cerebral blood flow (rCBF) with cognitive decline in Alzheimer’s disease (AD), no reports have simultaneously considered the effects of both factors on cognitive decline.

**Objective:**

The purpose of the present study was to compare WMH volume and rCBF in relation to cognitive function by developing a new software program to fuse magnetic resonance imaging (MRI) and single-photon emission computed tomography (SPECT) data.

**Method:**

We used MRI, SPECT, and neuropsychological data from 182 serial outpatients treated at the memory clinic of our hospital.

**Results:**

Twenty-nine AD patients fulfilled the inclusion criteria (18 females, mean age: 73.1 ± 7.9 years, mean Mini-Mental State Examination: 23.1 ± 3.0). Analysis of variance revealed that posterior deep WMH (DWMH) volume was significantly larger than both anterior periventricular hyperintensity (PVH) and DWMH, and posterior PVH volumes. Multivariate regression analysis showed that increased volumes of the anterior PVH and the posterior DWMH and decreased rCBF of the parietal cortex negatively affected cognitive function. The other areas had no significant negative effects on cognitive function.

**Conclusion:**

Our findings show that the volume of the posterior WMH was significantly larger than that of other areas, and the increased posterior WMH volume and decreased rCBF of the parietal cortex negatively affected cognitive function. Therefore, the posterior WMH volume and the parietal rCBF are key parameters of cognitive decline in AD patients.

## Introduction

White matter hyperintensities (WMHs) on T2-weighted fluid-attenuated inversion recovery (FLAIR) magnetic resonance imaging (MRI) sequences are linked to a risk of developing Alzheimer’s disease (AD) (1–4). WMHs have multiple histopathological correlates including demyelination, ependymal loss, cerebral ischemia, venous collagenosis, and microcystic infarcts and represent alterations in axon structure, gliosis, and small vessel disease (5–7). The longitudinal progression of WMH is associated with vascular risk factors such as aging (8), hypertension (9), and diabetes (10). In addition, WMHs modulate cognitive decline in AD (11–23).

Previous characterization of AD patients using single-photon emission computed tomography (SPECT) revealed decreased regional cerebral blood flow (rCBF) in the parietal, temporal, and posterior cingulate cortices (24–29). In this regard, SPECT has been useful for diagnosing AD (30). In addition, several studies have revealed age-related differences in rCBF and metabolism with hypoperfusion in the parietal lobe in early onset AD and medial temporal lobe in late-onset AD (31, 32).

While several studies support an association between the WMH volume or rCBF and cognitive decline in AD, no reports have simultaneously considered the effects of both WMH volume and rCBF on cognitive decline. Several methods have been proposed for performing quantitative white matter lesion load measurements on MRI (33–40). However, there is currently no software that allows fusion of MRI and SPECT data. The purpose of the present study was to compare WMH volume and rCBF in relation to cognitive function by using a software to fuse MRI and SPECT data. We hypothesized that WMH volume and rCBF have specific additive and independent effects on cognitive decline. We developed a novel software program to examine the relationship between WMH volume and cognitive function for different cognitive domains and used technetium-99m-ethyl cysteinate diethylester (^99m^Tc-ECD) SPECT to evaluate whether the relationship between WMH volume and cognitive function is independent of rCBF.

## Materials and Methods

### Participants

In accordance with the principles of the Declaration of Helsinki, we prospectively registered 182 serial patients who consulted the memory clinic of the Mie University Hospital. All procedures followed the clinical study guidelines of the ethics committee of the Mie University hospital and were approved by the internal review board. All procedures were described to the patients, and informed consent was obtained from them or their caregivers in the written form. Neurologists with sufficient experience in examining patients with dementia comprehensively examined each patient in our study. We collected data from the patients who fulfilled the following inclusion criteria: (1) patients who consulted the Memory Clinic of the Mie University Hospital from October 2013 to August 2016 and were diagnosed with AD based on pre-established criteria, fulfilling the criteria for probable AD of the National Institute of Neurologic Disorders and Stroke/Alzheimer Disease and Related Disorders Association (NINCDS-ADRDA) (41); (2) patients who received a neuroimaging examination on a 3T-MRI; (3) patients who received a ^99m^Tc-ECD SPECT examination; and (4) patients who underwent neuropsychological assessments. The exclusion criteria were as follows: (1) patients who were not examined using MRI and ^99^^m^Tc-ECD SPECT; (2) patients who were not administered neuropsychological assessments; (3) patients who were diagnosed with dementia other than AD; and (4) patients who had normal cognitive function.

### MRI Protocol and Evaluation of WMHs

Magnetic resonance imaging studies were performed using two different 3T-MRI scanners (Achieva and Ingenia; Philips Health Care, Best, the Netherlands). We used 3D-FLAIR and T1-weighted images. The parameters for 3D FLAIR were as follows: repetition time (TR), 6,000 ms; echo time (TE), 310 ms; inversion time, 2,000 ms; turbo factor, 203; sensitivity encoding factor, 3; field of view (FOV), 25 cm; matrix size, 480 × 256; and section thickness, 1.14 mm. The parameters for T1-weighted images were as follows: TR, 7.6 ms; TE, 3.6 ms; flip angle, 8°; FOV, 250 mm × 250 mm; in-plane resolution, 1.04 mm × 1.04 mm; and slice thickness, 0.7 mm.

Tissue quantification was performed using a novel in-house software (FUsed Software for Imaging Of Nervous system: FUSION) that yielded an individualized volumetric profile of brain tissue. The obtained T1-weighted and FLAIR images were imported from DICOM format files for processing. To increase the accuracy of segmentation, we used the Lesion Segmentation Tool for lesion filling (42). Lesion filling was applied to T1-weighted images that were in alignment with the lesion probability map. For the preprocessing level, T1-weighted images were coregistered to FLAIR images. Next, to separate WM, segmentation was performed by using the T1-weighted images and a mask of cerebral ventricles. The preprocessing function was based on SPM 8 (Wellcome Trust Centre for Neuroimaging, UCL). Second-level tissue segmentation was performed to separate WMHs from WM, using a semiautomated operation that extracted the pixels falling within predetermined value as WMHs. The WMH volume, which appeared as hyperintense areas on FLAIR images, was quantified for each area. The brain tissue was classified into four areas based on the division of the longitudinal fissure of the cerebrum and central sulcus. WMHs were automatically classified as periventricular hyperintensity (PVH) or deep WMH (DWMH), and their corrected volumes were calculated in cubic centimeters (cc).

### SPECT Protocol and Evaluation of rCBF

Intravenous radionuclide angiography was performed by bolus injection of the reconstituted 99mTc-ethyl cysteinate dimer (ECD) (600 MBq). Passage of the tracer from the aortic arch to the brain was monitored in a 128 × 128 format for 120 s at 1-s intervals using a three-head gamma camera system (GCA-9300A/DI, Toshiba, Tokyo, Japan) equipped with low-energy high-resolution fanbeam collimators. SPECT images were reconstructed by filtered back-projection using a ramp filter follower and postprocessing with a Butterworth filter. The triple-energy window technique was employed for scatter correction. ROIs were placed manually over the aortic arch and bilateral cerebral hemispheres. Time activity curves of these two ROIs were plotted and the brain perfusion index (BPI) was determined as described previously. BPI was then converted to the mean CBF (mCBF) value. The rCBF values were obtained by the conversion of total counts in brain SPECT into mCBF, using Lassen’s correction (43).

A three-dimensional stereotactic region of interest (ROI) template (3DSRT) program (FUJIFILM RI Pharma Co., Ltd.) was applied to assess the regional quantitative value (44–48). 3DSRT is a fully automated rCBF quantification program that can be used to examine a total of 636 ROIs. These ROIs are categorized into six brain segments on the 3DSRT template: bilateral parietal, temporal, and posterior cingulate cortices determined by previous studies (24–29). The blood flow to each ROI was quantified in mL/100 g/min.

**Figure 1:**
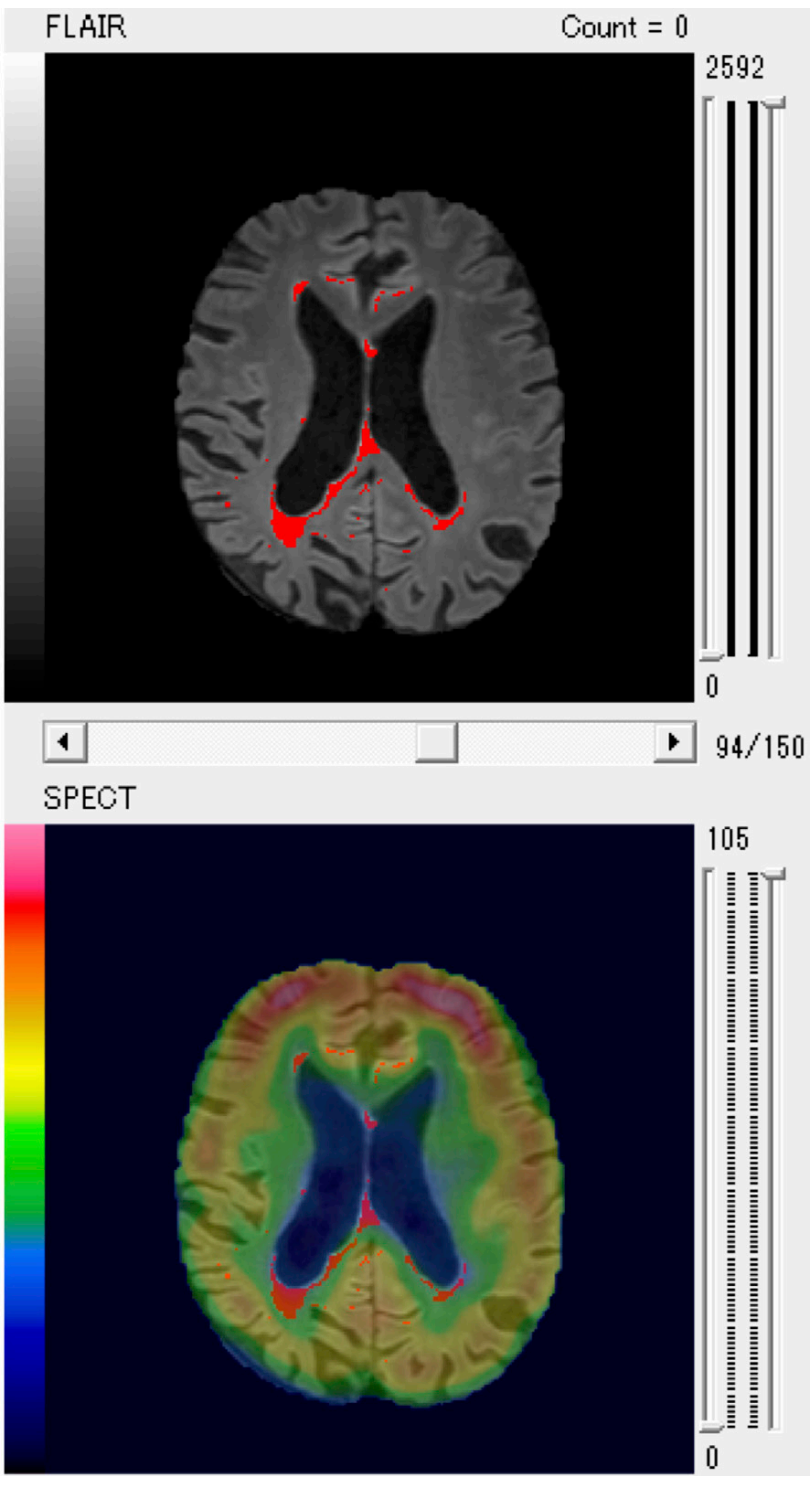
A patient with larger posterior white matter hyperintensity (WMH) volume and decreased regional cerebral blood flow of parietal region shown as a typical example of fused WMH and single-photon emission computed tomography (SPECT) images for quantitative volume analysis.

Finally, the results of evaluation of the SPECT images were fused with WMHs on MRI scans (Figure 1).

### Neuropsychological Assessments

The Mini-Mental State Examination (MMSE) (49) and Raven’s Colored Progressive Matrices (RCPM) (50) were used to quantify intellectual function. Memory was evaluated using the Rivermead Behavioural Memory Test (RBMT) (51). Assessment of visuospatial constructional ability was based on the method described by Strub and Black (52). A simple cube and a Necker cube were shown to the participants, and they were asked to draw them one by one. Each drawing was scored by assigning one of four possible grades (0: poor, 1: fair, 2: good, and 3: excellent). Frontal lobe function was assessed using two tasks, word fluency (WF) and the trail-making test A and B (TMT-A/-B) (53). The WF test consisted of category and letter domains. For the categorical WF, participants were asked to name as many animals as possible in 1 min. For the letter WF task, participants were asked to say the name of objects that begin with each of four phonemes, ka, sa, ta, and te (54).

### Statistical Analyses

Statistical analyses were performed with the Statistical Package for the Social Sciences, Version 20 (IBM Corp., Armonk, New York, NY, USA). Statistical analyses were conducted using one-way analysis of variance for continuous variables and the Ryan method for pairwise comparisons. We performed multivariate regression analysis using a generalized linear model. As predictor variable, we used age, and the PVH, DWMH, and CBFs of temporal, posterior cingulate, and parietal regions. As a response variable, we used MMSE, RCPM, RBMT, TMT-A/B, WF, and visuospatial constructional ability. A *p*-value of <0.05 was regarded as statistically significant.

## Results

### Clinical Data

A total of 182 patients were registered for the present study, and 29 patients fulfilled the inclusion criteria. Their clinical characteristics are shown in Table 1. The mean (±SD) age was 73.1 ± 7.9 years, and 18 of the participants were female. The mean (±SD) MMSE was 23.1 ± 3.0.

**Table 1:** Clinical characteristics.

	Mean (SD)	Prevalence *n* (%)
Age (years)	73.1 (7.9)	
Female		18 (62.1)
Education (years)	10.9 (2.6)	
Hypertension		12 (41.0)
Hyperlipidemia		6 (21.0)
Diabetes mellitus		4 (14.0)
MMSE	23.1 (3.0)	
RCPM (score)	24.4 (4.6)	
RCPM (time, s)	546.7 (322.4)	
RBMT (SPS)	8.6 (5.2)	
RBMT (SS)	3.3 (2.5)	
TMT A (s)	252.3 (118.9)	
WF (category, /min)	10.5 (3.7)	
WF (category, /min)	5.9 (2.8)	
Construction (cube)	2.4 (0.8)	
Construction (Necker cube)	2.2 (0.9)	

*MMSE, Mini-Mental State Examination; RBMT, Rivermead behavioral memory test; RCPM, Raven’s colored progressive matrices; WF, word fluency*.

### WMH Volume and rCBF

The participants’ WMH volumes and rCBF values are shown in Table 2 (a,b). The mean total WMH volume was 18.4 ± 19.3 cc. Analysis of variance [*F*(3,84) = 2.748, *p* < 0.05] and pairwise comparisons revealed that posterior DWMH volume was significantly larger than the anterior PVH (*p* = 0.038), anterior DWMH (*p* = 0.043), and posterior PVH volume (*p* = 0.009).

**Table 2:** Study participant WMH volumes (a) and rCBF values (b).

(a)	
Region (WMH)	Cubic centimeter [mean (SD)]
Total	18.4 (19.3)
PVH Right anterior	2.4 (1.9)
Right posterior	2.3 (1.3)
Left anterior	1.9 (1.8)
Left posterior	1.5 (1.2)
DWMH Right anterior	2.1 (3.3)
Right posterior	3.4 (3.9)
Left anterior	2.2 (3.3)
Left posterior	2.8 (4.5)

**(b)**	

**Region (rCBF)**	**Mean (SD)**

Callosomarginal (R)	37.2 (6.8)
Callosomarginal (L)	37.1 (6.8)
Precentral (R)	40.1 (6.9)
Precentral (L)	39.2 (6.6)
Central (R)	40.7 (7.5)
Central (L)	41.2 (7.0)
Parietal (R)	38.8 (7.1)
Parietal (L)	38.9 (7.0)
Angular (R)	42.8 (7.7)
Angular (L)	42.5 (7.4)
Temporal (R)	36.9 (6.7)
Temporal (L)	36.3 (6.1)
Occipital (R)	42.7 (7.1)
Occipital (L)	43.2 (6.9)
Pericallosal (R)	37.5 (7.3)
Pericallosal (L)	37.2 (7.1)
Lentiform nucleus (R)	36.9 (6.6)
Lentiform nucleus (L)	35.6 (5.6)
Thalamus (R)	32.4 (6.9)
Thalamus (L)	32.2 (6.2)
Hippocampus (R)	27.6 (4.9)
Hippocampus (L)	27.0 (4.8)
Cerebellum (R)	47.0 (8.7)
Cerebellum (L)	47.9 (8.4)
Posterior Cingulate (R)	42.1 (8.1)
Posterior Cingulate (L)	40.1 (7.4)

*WMH, white matter hyperintensity; PVH, periventricular hyperintensity; DWMH, deep WMH; rCBF, regional cerebral blood flow*.

### Association of WMH, rCBF, and Cognitive Decline

The effect of regional WMHs and rCBF on neuropsychological test results was evaluated with multivariate regression analysis (Table 3). The analysis showed that increased age correlated negatively with intellectual function (*p* = 0.008).

**Table 3:** Regional WMH effects on neuropsychological assessment results were evaluated with multivariate regression analysis.

	RCPM (score)	RCPM (time, s)	TMT A (s)	WF (category, /min)	Visuospatial constructional ability (Necker cube)
Intercept	20.58 (9.96−42.52)***	129.08 (17.70−941.53)***	644.34 (169.02−2456.32)***	14.20 (3.84−52.49)***	1.41 (0.29−6.81)
Age	1.00 (0.99−1.01)	1.03 (1.01−1.06)^**,a^	1.01 (0.99−1.03)	0.99 (0.98−1.01)	1.00 (0.98−1.02)
PVH (A)	0.97 (0.89−1.06)	1.00 (0.79−1.26)	1.17 (0.98−1.40)	1.08 (0.93−1.25)	0.80 (0.66−0.96)^*,a^
PVH (P)	1.01 (0.92−1.11)	0.96 (0.73−1.28)	0.87 (0.70−1.08)	1.05 (0.88−1.24)	1.02 (0.85−1.22)
DWMH (A)	1.06 (1.00−1.13)	1.05 (0.90−1.21)	0.91 (0.81−1.03)	1.02 (0.92−1.13)	1.17 (0.96−1.41)
DMWH (P)	0.97 (0.93−1.01)	0.98 (0.88−1.09)	1.08 (0.99−1.17)	0.89 (0.83−0.96)^**,a^	0.96 (0.85−1.09)
CBF/parietal	1.01 (0.99−1.04)	0.94 (0.89−1.00)^*,a^	0.92 (0.88−0.96)^***,a^	0.98 (0.95−1.02)	1.08 (1.02−1.14)^**,a^
CBF/temporal	1.02 (0.99−1.05)	1.01 (0.94−1.09)	1.05 (1.00−1.11)	1.01 (0.96−1.06)	0.94 (0.88−1.00)
CBF/posterior cingulate	0.98 (0.96−1.00)**	1.04 (0.98−1.09)	0.99 (0.95−1.03)	1.01 (0.98−1.05)	0.99 (0.93−1.06)

*Odds ratio (95% CI)*.

*p < 0.05.

**p < 0.01.

***p < 0.001.

*^a^Negative effect*.

*A, anterior; P, posterior; PVH, periventricular hyperintensity; DWMH, deep white matter hyperintensity; MMSE, Mini-Mental State Examination; RCPM, Raven’s Coloured Progressive Matrices; RBMT, Rivermead Behavioural Memory Test; SPS, Standardized Profile Score; SS, screening score; TMT, trail making test; WF, word fluency*.

Multivariate regression analysis also showed that WMHs and rCBF were significantly associated with memory and intellectual, frontal, and visuospatial functions, as follows. An increased anterior PVH volume negatively affected visuospatial constructional ability (*p* = 0.016) and increased posterior DWMH volume negatively affected WF (category), whereas increased volumes in other areas did not have negative impacts on cognitive function. In addition, decreased rCBF in the parietal areas negatively affected RCPM (time) (*p* = 0.033), TMT-A (*p* < 0.001), and visuospatial constructional ability (*p* = 0.012), whereas decreased rCBF in other areas did not.

In particular, the anterior PVH, the posterior DWMH, and the parietal regions were key areas that negatively affected cognitive function. We present a representative patient with increased posterior WMH volume and decreased rCBF of the parietal region as a typical example in Figure 1.

### Summarize the Results

We compared MRI and SPECT findings with neuropsychological data from outpatients of the memory clinic of our hospital. An analysis of variance revealed that the posterior DWMH volume was significantly larger than the anterior PVH, anterior DWMH, and posterior PVH volumes. Multivariate regression analysis showed that increased anterior PVH and left posterior DWMH volumes and decreased rCBF of the parietal area correlated negatively with cognitive function, whereas other areas did not have negative effects on cognitive function.

## Discussion

We developed a novel software program for quantitative volume analysis of WMHs and SPECT using fused imaging data. Our software has some advantages over other programs and showed reasonable results in accordance with previous studies. Although several software programs have been developed for WMH analysis (39, 40), none are available for discrimination of PVH and DWMH on the anatomical basis of blood flow cliff or for the combined evaluation of rCBF. Measuring white matter volume is critical for the diagnosis of dementia, and fusion images of WMHs and rCBF are also useful in clinical settings. In addition to these regional analyses, we examined brain tissue after classification into four areas based on division of the longitudinal fissure of the cerebrum and central sulcus. Other anatomical ROIs have also been used (55); nonetheless, the regions we investigated may be useful in practical settings.

Previous studies have reported that the WMH posterior volume was larger than the anterior volume (56–58) in patients with cerebral amyloid angiopathy (CAA) as well as AD (59). CAA induces cerebral hypoperfusion in the white matter as a result of amyloid β deposition in the microvessels of the cerebral cortices and is found in more than 80% of all patients with AD (60). It is, therefore, likely that most patients in our cohort had comorbid CAA. Our results showed that the posterior WMH volume, especially that of the DWMH, was larger than the anterior and posterior PVH volumes. The results of the present study are in agreement with those of previous studies and further indicate that posterior volume, especially posterior DWMH, may be a key characteristic of AD.

The present study showed that WMHs were significantly associated with cognitive functions, which is in accordance with previous findings (11–23). Additionally, our results indicated that increased volumes of the anterior PVH and posterior DWMH negatively affected frontal and visuospatial function, both of which decline in AD (33). Given the larger posterior WMH volume, it is reasonable to hypothesize that posterior WMH negatively affected cognitive function in AD. On the other hand, SPECT results indicated that the parietal region more negatively affected cognitive function than did other regions. The area also negatively affected intellectual, frontal, and visuospatial function. A previous study showed that distinct cognitive profiles are associated with anterior and posterior WMH progression (61), When simultaneously considering the effects of both WMHs and rCBF factors (as shown in Figure 1), modulation of posterior regions may underscore neurodegeneration in the posterior association cortex and cognitive decline in AD. While increased WMH volume and decreased rCBF negatively affected cognitive function, some areas were associated with improvement in certain cognitive domains.

The present study has several limitations, including the small number of patients, investigating only patients with AD, and a lack of gray matter volume measurements. The patient cohort was small due to the prohibitive cost of SPECT imaging and standardized data collection form ECD as a radiolabeled ligand. Most patients in our cohort had AD, preventing us from establishing a significant effect of pathological background. Besides patients with AD, information is lacking in other types of dementias. Future studies that investigate other types of dementias, as well as gray and white matter volumes, could advance WMH and rCBF analysis to predict cognitive decline more accurately.

## Conclusion

Our results agreed with those of previous studies, indicating that our software is a reliable tool. Collectively, the existing evidence suggests that posterior WMH volume and parietal cortex rCBF may predict cognitive decline in AD.

## Ethics Statement

In accordance with the principles of the Declaration of Helsinki, we prospectively registered 182 serial patients who consulted the memory clinic of the Mie University Hospital. All procedures followed the clinical study guidelines of the ethics committee of the Mie University hospital and were approved by the internal review board. All procedures were described to the patients, and informed consent was obtained from them or their caregivers.

## Author Contributions

HK, MS, and HT conceived and designed the experiments. KT, HK, and MS performed the experiments. TH developed the software. KT analyzed the data. KT, MS, and HT wrote the paper.

## Conflict of Interest Statement

The Department of Dementia Prevention and Therapeutics, at Mie University Graduate School of Medicine is an endowment department supported by grants from FUJIFILM RI Pharma Co., Ltd., and Ise keiyu Hospital.

## References

[1] DeCarli C Miller BL Swan GE Reed T Wolf PA Carmelli D. Cerebrovascular and brain morphologic correlates of mild cognitive impairment in the National Heart, Lung, and Blood Institute Twin Study. Arch Neurol (2001) 58(4):643–7. 10.1001/archneur.58.4.643 11295996

[2] Nordahl CW Ranganath C Yonelinas AP Decarli C Fletcher E Jagust WJ. White matter changes compromise prefrontal cortex function in healthy elderly individuals. J Cogn Neurosci (2006) 18(3):418–29. 10.1162/jocn.2006.18.3.418 16513006 PMC3776596

[3] Yoshita M Fletcher E Harvey D Ortega M Martinez O Mungas DM Extent and distribution of white matter hyperintensities in normal aging, MCI, and AD. Neurology (2006) 67(12):2192–8. 10.1212/01.wnl.0000249119.95747.1f 17190943 PMC3776588

[4] Brickman AM Provenzano FA Muraskin J Manly JJ Blum S Apa Z Regional white matter hyperintensity volume, not hippocampal atrophy, predicts incident Alzheimer disease in the community. Arch Neurol (2012) 69:1621–7. 10.1001/archneurol.2012.1527 22945686 PMC3597387

[5] Kim K Macfall J Payne M. Classification of white matter lesions on magnetic resonance imaging in elderly persons. Biol Psychiatry (2008) 64(4):273–80. 10.1016/j.biopsych.2008.03.024 18471801 PMC2593803

[6] Gouw AA Seewann A van der Flier WM Barkhof F Rozemuller AM Scheltens P Heterogeneity of small vessel disease: a systematic review of MRI and histopathology correlations. J Neurol Neurosurg Psychiatry (2011) 82(2):126–35. 10.1136/jnnp.2009.204685 20935330

[7] Schmidt R Schmidt H Haybaeck J Loitfelder M Weis S Cavalieri M Heterogeneity in age-related white matter changes. Acta Neuropathol (2011) 122(2):171–85. 10.1007/s00401-011-0851-x 21706175

[8] Vannorsdall TD Waldstein SR Kraut M Pearlson GD Schretlen DJ. White matter abnormalities and cognition in a community sample. Arch Clin Neuropsychol (2009) 24(3):209–17. 10.1093/arclin/acp037 19617597 PMC2765350

[9] Wolfson L Wakefield DB Moscufo N Kaplan RF Hall CB Schmidt JA Rapid buildup of brain white matter hyperintensities over 4 years linked to ambulatory blood pressure, mobility, cognition and depression in old persons. J Gerontol A Biol Sci Med Sci (2013) 68(11):1387–94. 10.1093/gerona/glt072 23766429 PMC3805298

[10] Taylor WD MacFall JR Provenzale JM Payne ME McQuoid DR Steffens DC Serial MR imaging of volumes of in elderly patients: correlation with vascular risk factors. Am J Roentgenol (2003) 181(2):571–6. 10.2214/ajr.181.2.1810571 12876050

[11] Defrancesco M Marksteiner J Deisenhammer E Kemmler G Djurdjevic T Schocke M. Impact of white matter lesions and cognitive deficits on conversion from mild cognitive impairment to Alzheimer’s disease. J Alzheimers Dis (2013) 34(3):665–72. 10.3233/JAD-122095 23254639

[12] Kimura N Nakama H Nakamura K Aso Y Kumamoto T. Relationship between white matter lesions and progression of cognitive decline in Alzheimer’s disease. Dement Geriatr Cogn Dis Extra (2013) 3(1):96–101. 10.1159/000350317 23637702 PMC3638977

[13] DeCarli C Murphy DG Tranh M Grady CL Haxby JV Gillette JA The effect of white matter hyperintensity volume on brain structure, cognitive performance, and cerebral metabolism of glucose in 51 healthy adults. Neurology (1995) 45(11):2077–84. 10.1212/WNL.45.11.2077 7501162

[14] De Groot JC De Leeuw FE Oudkerk M Van Gijn J Hofman A Jolles J Periventricular cerebral white matter lesions predict rate of cognitive decline. Ann Neurol (2002) 52(3):335–41. 10.1002/ana.10294 12205646

[15] Buckner RL. Memory and executive function in aging and AD: multiple factors that cause decline and reserve factors that compensate. Neuron (2004) 44(1):195–208. 10.1016/j.neuron.2004.09.006 15450170

[16] Longstreth WT Jr Arnold AM Beauchamp NJ Jr Manolio TA Lefkowitz D Jungreis C Incidence, manifestations and predictors of worsening white matter on serial cranial magnetic resonance imaging in the elderly: the cardiovascular health study. Stroke (2005) 36(1):56–61. 10.1161/01.STR.0000149625.99732.69 15569873

[17] Kramer JH Mungas D Reed BR Wetzel ME Burnett MM Miller BL Longitudinal MRI and cognitive change in healthy elderly. Neuropsychology (2007) 21(4):412–8. 10.1037/0894-4105.21.4.412 17605574 PMC2780018

[18] Stewart R Dufouil C Godin O Ritchie K Maillard P Delcroix N Neuroimaging correlates of subjective memory deficits in a community population. Neurology (2008) 70(18):1601–7. 10.1212/01.wnl.0000310982.99438.54 18443310

[19] Carmichael O Schwarz C Drucker D Fletcher E Harvey D Beckett L Longitudinal changes in white matter disease and cognition in the first year of the Alzheimer disease neuroimaging initiative. Arch Neurol (2010) 67(11):1370–8. 10.1001/archneurol.2010.284 21060014 PMC3082636

[20] Debette S Seshadri S Beiser A Au R Himali JJ Palumbo C Midlife vascular risk factor exposure accelerates structural brain aging and cognitive decline. Neurology (2011) 77(5):461–8. 10.1212/WNL.0b013e318227b227 21810696 PMC3146307

[21] Akisaki T Sakurai T Takata T Umegaki H Araki A Mizuno S Cognitive dysfunction associates with white matter hyperintensities and subcortical atrophy on magnetic resonance imaging of the elderly diabetes mellitus Japanese elderly diabetes intervention trial (J-EDIT). Diabetes Metab Res Rev (2006) 22(5):376–84. 10.1002/dmrr.632 16506272

[22] Maillard P Carmichael O Fletcher E Reed B Mungas D DeCarli C. Coevolution of white matter hyperintensities and cognition in the elderly. Neurology (2012) 79(5):442–8. 10.1212/WNL.0b013e3182617136 22815562 PMC3405254

[23] Sonohara K Kozaki K Akishita M Nagai K Hasegawa H Kuzuya M White matter lesions as a feature of cognitive impairment, low vitality and other symptoms of geriatric syndrome in the elderly. Geriatr Gerontol Int (2008) 8(2):93–100. 10.1111/j.1447-0594.2008.00454.x 18713161

[24] Drzezga A Lautenschlager N Siebner H Riemenschneider M Willoch F Minoshima S Cerebral metabolic changes accompanying conversion of mild cognitive impairment into Alzheimer’s disease: a PET follow-up study. Eur J Nucl Med Mol Imaging (2003) 30(8):1104–13. 10.1007/s00259-003-1194-1 12764551

[25] Minoshima S Giordani B Berent S Frey KA Foster NL Kuhl DE. Metabolic reduction in the posterior cingulate cortex in very early Alzheimer’s disease. Ann Neurol (1997) 42(1):85–94. 10.1002/ana.410420114 9225689

[26] Huang C Wahlund LO Svensson L Winblad B Julin P. Cingulate cortex hypoperfusion predicts Alzheimer’s disease in mild cognitive impairment. BMC Neurol (2002) 2(1):9. 10.1186/1471-2377-2-9 12227833 PMC128832

[27] Johnson KA Moran EK Becker JA Blacker D Fischman AJ Albert MS. Single photon emission computed tomography perfusion differences in mild cognitive impairment. J Neurol Neurosurg Psychiatry (2007) 78(3):240–7. 10.1136/jnnp.2006.096800 17056633 PMC2117661

[28] Borroni B Anchisi D Paghera B Vicini B Kerrouche N Garibotto V Combined 99mTc-ECD SPECT and neuropsychological studies in MCI for the assessment of conversion to AD. Neurobiol Aging (2006) 27(1):24–31. 10.1016/j.neurobiolaging.2004.12.010 16298237

[29] Dougall NJ Bruggink S Ebmeier KP. Systematic review of the diagnostic accuracy of 99mTc-HMPAO-SPECT in dementia. Am J Geriatr Psychiatry (2004) 12(6):554–70. 10.1097/00019442-200411000-00002 15545324

[30] Holman BL Johnson KA Gerada B Carvalho PA Satlin A The scintigraphic appearance of Alzheimer’s disease: a prospective study using technetium-99m-HMPAO SPECT. J Nucl Med (1992) 33(2):181–5. 1732438

[31] Kemp PM Holmes C Hoffmann SM Bolt L Holmes R Rowden J Alzheimer’s disease: differences in technetium-99m HMPAO SPECT scan findings between early onset and late onset dementia. J Neurol Neurosurg Psychiatry (2003) 74(6):715–9. 10.1136/jnnp.74.6.715 12754337 PMC1738480

[32] Hanyu H Shimizu S Tanaka Y Takasaki M Koizumi K Abe K Effect of age on regional cerebral blood flow patterns in Alzheimer’s disease patients. J Neurol Sci (2003) 209(102):25–30. 10.1016/S0022-510X(02)00456-2 12686398

[33] Grimaud J Lai M Thorpe J Adeleine P Wang L Barker GJ Quantification of MRI lesion load in multiple sclerosis: a comparison of three computer-assisted techniques. Magn Reson Imaging (1996) 14(5):495–505. 10.1016/0730-725X(96)00018-5 8843362

[34] Kikinis R Guttmann C Metcalf D Wells WM III Ettinger GJ Weiner HL Quantitative follow-up of patients with multiple sclerosis using MRI: technical aspects. J Magn Reson Imaging (1999) 9(4):519–30. 10.1002/(SICI)1522-2586(199904) 10232509

[35] Alfano B Brunetti A Larobina M Quarantelli M Tedeschi E Ciarmiello A Automated segmentation and measurement of global white matter lesion volume in patients with multiple sclerosis. J Magn Reson Imaging (2000) 12(6):799–807. 10.1002/1522-2586(200012) 11105017

[36] Itti L Chang L Ernst T. Segmentation of progressive multifocal leukoencephalopathy lesions in fluid-attenuated inversion recovery magnetic resonance imaging. J Neuroimaging (2001) 11(4):412–7. 10.1111/j.1552-6569.2001.tb00071.x 11677882

[37] Zijdenbos AP Forghani R Automatic “pipeline” analysis of 3-D MRI data for clinical trials: application to multiple sclerosis. IEEE Trans Med Imaging (2002) 21(10):1280–91. 10.1109/TMI.2002.806283 12585710

[38] Anbeek P Vincken KL Osch M Bisschops R van der Grond J. Probabilistic segmentation of white matter lesions in MR imaging. Neuroimage (2004) 21(3):1037–44. 10.1016/j.neuroimage.2003.10.012 15006671

[39] Admiraal-Behloul F Van den Heuvel DM Olofsen H Van Osch MJ Van der Grond J Van Buchem MA Fully automatic segmentation of white matter hyperintensities in MR images of the elderly. Neuroimage (2005) 28:607–17. 10.1016/j.neuroimage.2005.06.061 16129626

[40] Ambarki K Wåhlin A Birgander R Eklund A Malm J MR imaging of brain volumes: evaluation of a fully automatic software. AJNR Am J Neuroradiol (2011) 32(2):408–12. 10.3174/ajnr.A2275 21051511 PMC7965732

[41] McKhann G Drachman D Folstein M Katzman R Price D Stadlan EM Clinical diagnosis of Alzheimer’s disease: report of the NINCDS-ADRDA Work Group under the auspices of Department of Health and Human Services Task Force on Alzheimer’s Disease. Neurology (1984) 34(7):939–44. 10.1212/WNL.34.7.939 6610841

[42] Schmidt P Gaser C Arsic M Buck D Forschler A Berthele A An automated tool for detection of FLAIR-hyperintense white-matter lesions in multiple sclerosis. Neuroimage (2012) 59(4):3774–83. 10.1016/j.neuroimage.2011.11.032 22119648

[43] Lassen NA Andersen AR Friberg L Paulson OB. The retention of [99mTc]-d,l-HM-PAO in the human brain after intracarotid bolus injection: a kinetic analysis. J Cereb Blood Flow Metab (1988) 8:S13–22. 10.1038/jcbfm.1988.28 3192638

[44] Matsuda H Tsuji S Shuke N Sumiya H Tonami N Hisada K. A quantitative approach to technetium-99m hexamethylpropylene amine oxime. Eur J Nucl Med (1992) 19:195–200. 10.1007/BF00173281 1572384

[45] Matsuda H Yagishita A Tsuji S Hisada K. A quantitative approach to technetium-99m ethyl cysteinate dimer: a comparison with technetium-99m hexamethylpropylene amine oxime. Eur J Nucl Med (1995) 22:633–7. 10.1007/BF01254564 7498224

[46] Kobayashi S Tateno M Utsumi K Takahashi A Saitoh M Morii H Quantitative analysis of brain perfusion SPECT in Alzheimer’s disease using a fully automated regional cerebral blood flow quantification software, 3DSRT. J Neurol Sci (2008) 264(1–2):27–33. 10.1016/j.jns.2007.07.015 17764699

[47] Takeuchi R Matsuda H Yoshioka K Yonekura Y Cerebral blood flow SPECT in transient global amnesia with automated ROI analysis by 3DSRT. Eur J Nucl Med Mol Imaging (2004) 31(4):578–89. 10.1007/s00259-003-1406-8 14722677

[48] Takeuchi R Sengoku T Matsumura K. Usefulness of fully automated constant ROI analysis software for the brain: 3DSRT and FineSRT. Radiat Med (2006) 24:538–44. 10.1007/s11604-006-0054-x 17058151

[49] Folstein MF Folstein SE McHugh PR ‘Mini-mental state’. A practical method for grading the cognitive state of patients for the clinician. J Psychiatr Res (1975) 12:189–98. 10.1016/0022-3956(75)90026-6 1202204

[50] Raven JC Coloured Progressive Matrices Sets A, Ab, B. Manual Sections I and II. Oxford: Oxford Psychologists Press (1947).

[51] Wilson B Cockburn J Baddeley A The Rivermead Behavioural Memory Test. Bury St Edmunds, Suffolk: Valley Test Company (1985).

[52] Strub RL Black FW The Mental Status Examination in Neurology. 4th ed Philadelphia, PA: DAVIS Company (2001).

[53] Partington JE Leiter RG Partington’s Pathway Test. Psychol Serv Cent Bull (1949) 1:9–20.

[54] Dohi N Iwaya T Kayamori R Seishin-Kinou H The Evaluation of Mental Function (in Japanese). Tokyo: Ishiyaku Publishers, Inc. (1992).

[55] Wakana S Jiang H Nagae-Poetscher LM van Zijl PC Mori S. Fiber tract-based atlas of human white matter anatomy. Radiology (2004) 230(1):77–87. 10.1148/radiol.2301021640 14645885

[56] Thanprasertsuk S Martinez-Ramirez S Pontes-Neto OM Ni J Ayres A Reed A Posterior white matter disease distribution as a predictor of amyloid angiopathy. Neurology (2014) 83(9):794–800. 10.1212/WNL.0000000000000732 25063759 PMC4155043

[57] Vinters HV Gilbert JJ. Cerebral amyloid angiopathy: incidence and complications in the aging brain. II. The distribution of amyloid vascular changes. Stroke (1983) 14(6):924–8. 10.1161/01.STR.14.6.924 6658996

[58] Rosand J Muzikansky A Kumar A Wisco JJ Smith EE Betensky RA Spatial clustering of hemorrhages in probable cerebral amyloid angiopathy. Ann Neurol (2005) 58(3):459–62. 10.1002/ana.20596 16130107

[59] Holland CM Smith EE Csapo I Gurol ME Brylka DA Killiany RJ Spatial distribution of white-matter hyperintensities in Alzheimer disease, cerebral amyloid angiopathy, and healthy aging. Stroke (2008) 39(4):1127–33. 10.1161/STROKEAHA.107.497438 18292383 PMC2754400

[60] Tomimoto H. White matter integrity and cognitive dysfunction: radiological and neuropsychological correlations. Geriatr Gerontol Int (2015) 15(Suppl 1):3–9. 10.1111/ggi.12661 26671151

[61] Marquine MJJ Attix DK Goldstein LB Samsa GP Payne ME Chelune GJ Differential patterns of cognitive decline in anterior and posterior white matter hyperintensity progression. Stroke (2010) 41(9):1946–50. 10.1161/STROKEAHA.110.587717 20651266 PMC3279727

